# Author Correction: Single-cell profiling defines the prognostic benefit of CD39^high^ tissue resident memory CD8+ T cells in luminal-like breast cancer

**DOI:** 10.1038/s42003-021-02789-5

**Published:** 2021-10-27

**Authors:** Agnese Losurdo, Caterina Scirgolea, Giorgia Alvisi, Jolanda Brummelman, Valentina Errico, Luca Di Tommaso, Karolina Pilipow, Federico Simone Colombo, Bethania Fernandes, Clelia Peano, Alberto Testori, Corrado Tinterri, Massimo Roncalli, Armando Santoro, Emilia Maria Cristina Mazza, Enrico Lugli

**Affiliations:** 1 Laboratory of Translational Immunology, IRCCS Humanitas Research Hospital, Rozzano, Milan Italy; 2 Medical Oncology and Hematology Unit, IRCCS Humanitas Research Hospital, Rozzano, Milan Italy; 3 Breast Surgery Unit, IRCCS Humanitas Research Hospital, Rozzano, Milan Italy; 4 Department of Pathology, IRCCS Humanitas Research Hospital, Rozzano, Milan Italy; 5 Department of Biomedical Sciences, Humanitas University, Pieve Emanuele, Milan Italy; 6 Humanitas Flow Cytometry Core, IRCCS Humanitas Research Hospital, Rozzano, Milan Italy; 7 Genomic Unit, IRCCS Humanitas Research Hospital, Rozzano, Milan Italy; 8 Institute of Genetic and Biomedical Research, UoS Milan, National Research Council, Rozzano, Milan Italy

**Keywords:** Immunological memory, Breast cancer

Correction to: *Communications Biology* 10.1038/s42003-021-02595-z, published online 22 September 2021.

In this article, the institute “IRCCS Humanitas Research Hospital” was incorrectly given as “IRCSS Humanitas Research Hospital” in the following affiliations. The original article has been corrected.

Laboratory of Translational Immunology, IRCCS Humanitas Research Hospital, Rozzano, Milan, Italy.

Medical Oncology and Hematology Unit, IRCCS Humanitas Research Hospital, Rozzano, Milan, Italy.

Breast Surgery Unit, IRCCS Humanitas Research Hospital, Rozzano, Milan, Italy.

Department of Pathology, IRCCS Humanitas Research Hospital, Rozzano, Milan, Italy.

Humanitas Flow Cytometry Core, IRCCS Humanitas Research Hospital, Rozzano, Milan, Italy.

Genomic Unit, IRCCS Humanitas Research Hospital, Rozzano, Milan, Italy.

