# Supplementary material for: Single-cell profiling defines the prognostic benefit of CD39high tissue resident memory CD8+ T cells in luminal-like breast cancer
Source: Commun Biol. 2021 Sep 22;4:1117. doi: 10.1038/s42003-021-02595-z (PMC8458450; doi:10.1038/s42003-021-02595-z)
Supplement: Supplementary file 2 — Supplementary Information [file 42003_2021_2595_MOESM2_ESM.pdf]

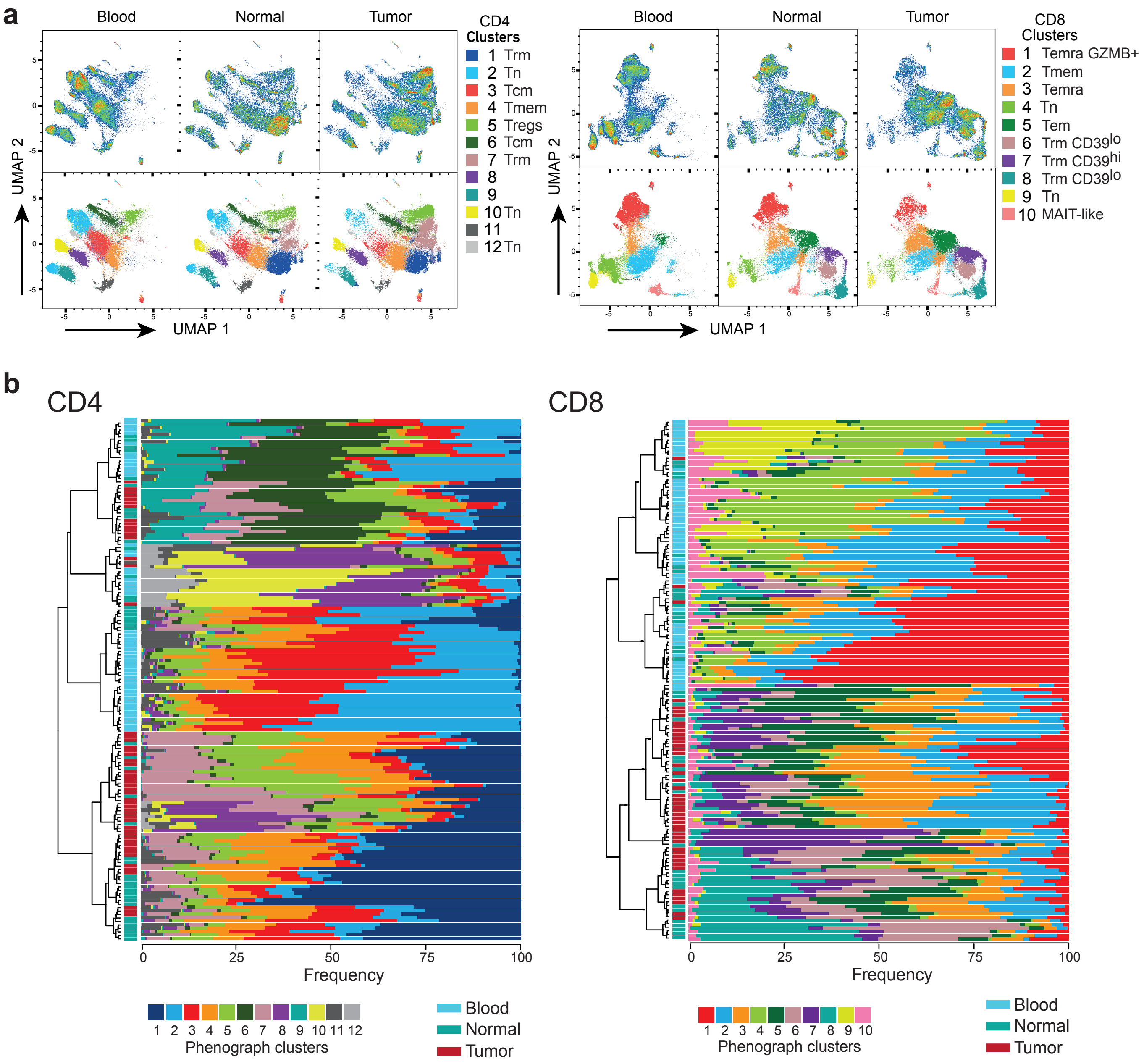

Supplementary Figure 1. Peripheral blood, breast tissue and tumor samples from BC patients display a different T cell immune composition. **a.** UMAP analysis of concatenated CD4+ and CD8+ T cells (1,000 cells/sample) from peripheral blood, normal breast tissue and tumor samples from 54 early breast cancer patients (top rows), and color-coded UMAP depicting clusters identified by PhenoGraph (bottom rows). **b.** Hierarchical metaclustering of the frequencies of the 12 CD4+ and 10 CD8+ T cell PhenoGraph clusters, identified as in Figure 1B, from single peripheral blood, normal breast tissue, and tumor samples.

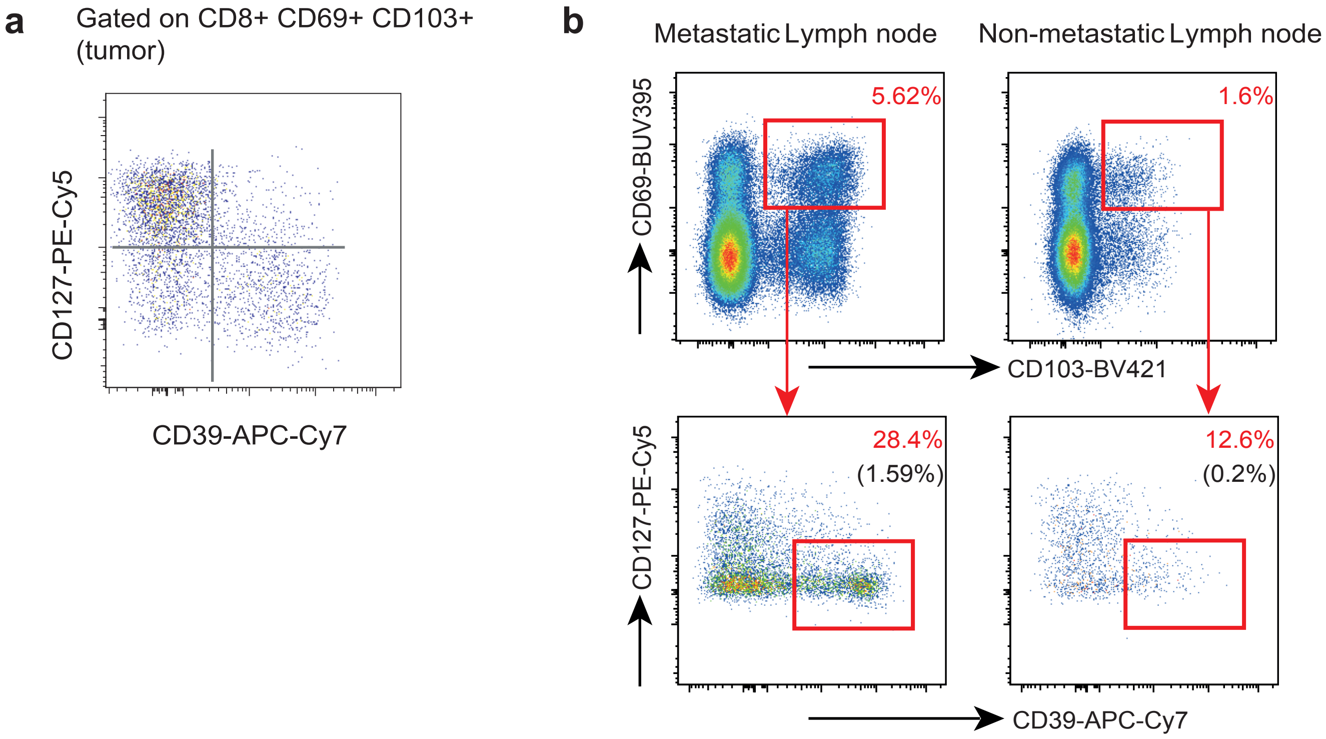

Supplementary Figure 2. BC tumors and metastatic lymph nodes show enrichment of CD127- CD39hi CD8+ Trm cells. **a.** Representative flow cytometry plot showing the different Trm populations identified by CD127 and CD39 expression from a BC tumor. **b.** Representative flow cytometric analysis showing the frequency of CD127- CD39hi among CD69+ CD103+ Trm from metastatic and non-metastatic lymph node samples. Numbers in the dot plots indicate the percentage of cells identified by the gates while those in brackets indicate the percentage of CD127- CD39hi Trm among total CD8+ cells. Cumulative data from the entire cohort are shown in Fig. 2D.

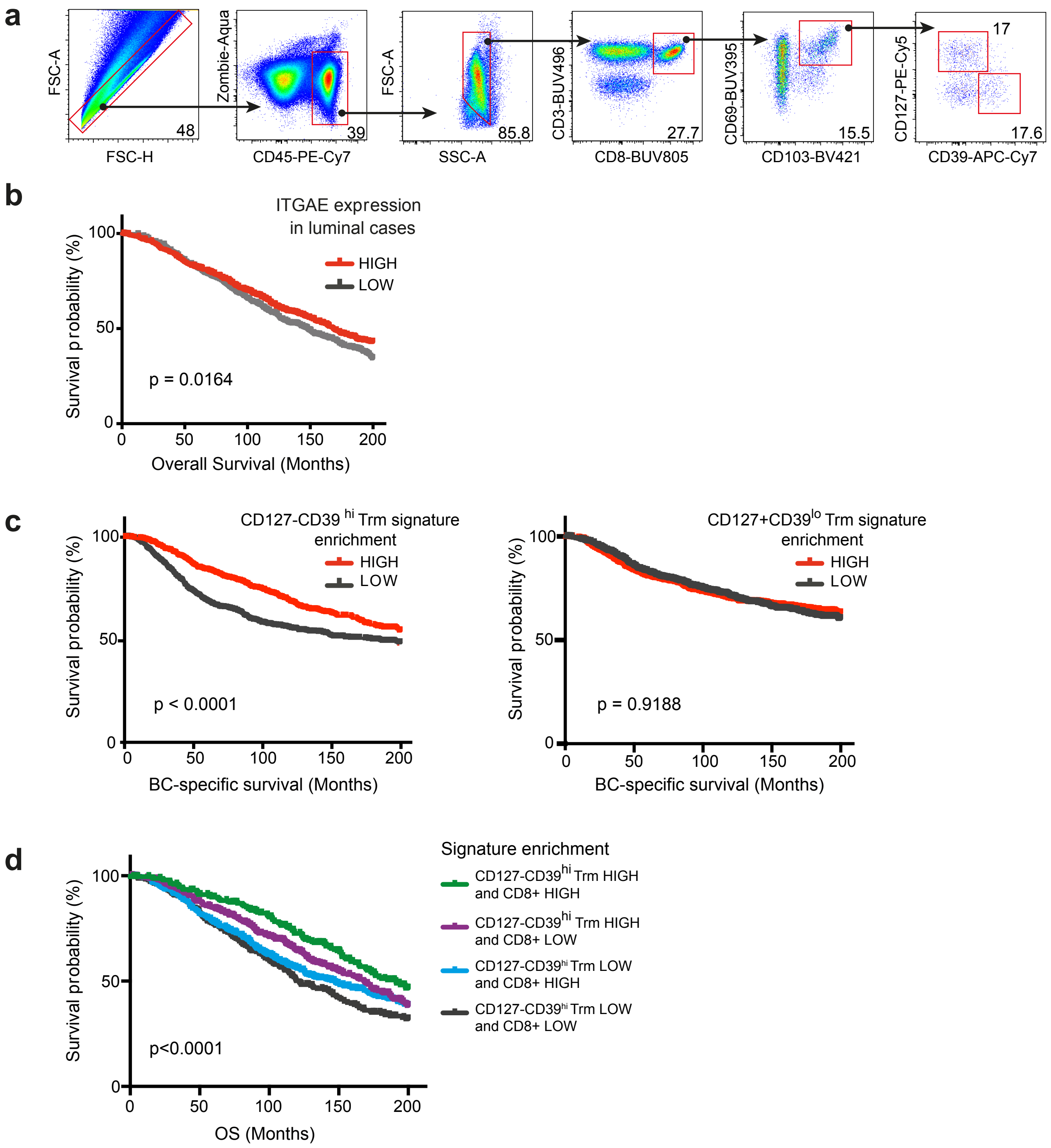

Supplementary Figure 3. Identification of CD8+ Trm infiltrating BC with differential expression of CD127 and CD39. **a.** Flow cytometric gating strategy for the identification of CD127+ CD39lo and CD127- CD39hi within CD69+ CD103+ CD8+ Trm cells used for cell sorting. Numbers in the dot plots indicate the percentage of cells identified by the gates. **b.** Kaplan-Meyer OS curves for luminal-like BC cases in the METABRIC consortium data set (n=1,436) according to high or low expression of ITGAE. **c.** Kaplan-Meier BC-specific survival curves in the METABRIC consortium data set (n=1,894). In B and C, groups were defined and data were displayed as in Fig. 3C. **d.** Kaplan-Meier overall survival (OS) curves in the METABRIC consortium data set in luminal-like BCs (n=1,436) according to high or low enrichment of CD127- CD39hi Trm, integrated with CD8A expression. The mean z-score value was used to classify tumor samples into LOW and HIGH expression groups. P values were calculated applying the Log-rank (Mantel-Cox) test.

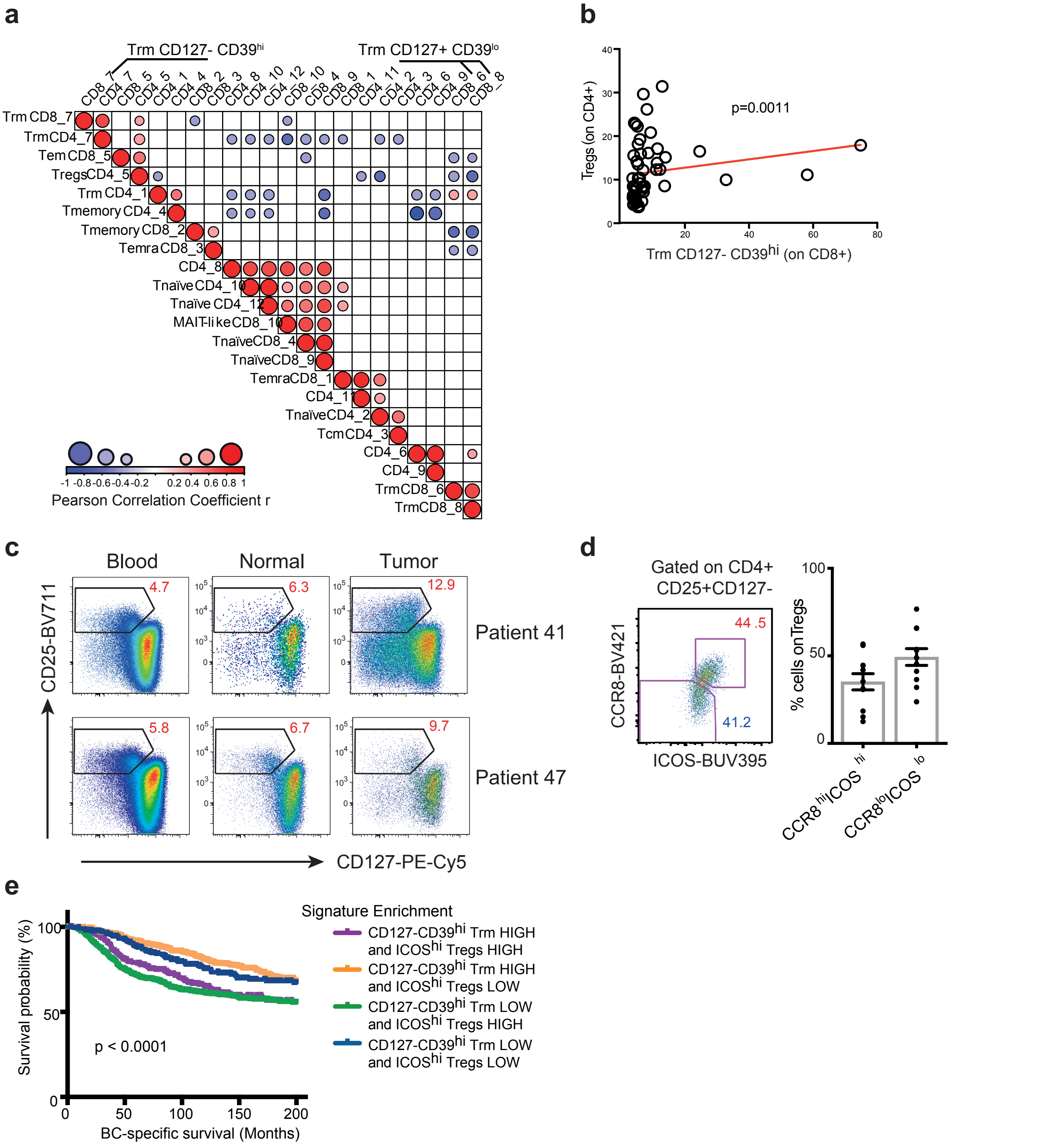

Supplementary Figure 4. CD4+ Treg and CD8+ Trm subset relationship in BC. a. Correlogram showing Pearson correlation between frequencies of CD4+ (T4) and CD8+ (T8) PhenoGraph clusters (obtained as in Fig. 1B) in tumor samples from BC patients (n=47 biologically independent samples). Non-significant correlations (p>0.05) were left blank. **b.** Spearman correlation between frequencies of intratumoral CD127- CD39hi Trm among total CD8+ and CD127- CD25+ Treg cells among total CD4+ as determined by manual gating (r=0.4369, p=0.0011; n= 47). **c.** Representative gating strategy for the isolation of CD127- CD25+ Treg cells in different tissue specimens from two patients. **d.** Representative gating strategy for the identification $CCR8^{hi}$ $ICOS^{hi}$ and $CCR8^{lo}$ $ICOS^{lo}$ Tregs in a tumor and summary plot of data from n=11 patients (mean±SEM; 3 independent experiments). **e.** Kaplan-Meier BC-specific survival curves in the METABRIC consortium data set (n=1,894)**.** Groups were defined and data were displayed as in Fig. 4f, g. In c and d, numbers in the flow cytometry plots indicate the percentage of cells identified by the gate.

Supplementary Table 1. Fluorescently-conjugated monoclonal antibodies used in the study

Specificity	Fluorochrome	Clone	Manufacturer	Cat. #	Lot. #	Panel
TCRgd	PerCP-Cy5.5	B1	BioLegend	331224	B260893	27-parameter
NKG2A	FITC	REA110	Miltenyi	130-113-568	5190115118	27-parameter
CD39	APC-H7	A1	BioLegend	328226	B260740	27-parameter
GZMB	APC-R700	GB11	BD	560213	7339825	27-parameter
TIGIT	APC	A15153G	BioLegend	372706	B259593	27-parameter
CD25	BV786	2A3	BD	741035	9011676	27-parameter
CCR7	BV711	150503	BD	566602	8248691	27-parameter
OX40	BV650	ACT35	BD	563658	8288968	27-parameter
CD161	BV605	HP-3G10	BioLegend	339916	B245752	27-parameter
CD27	BV570	O323	BioLegend	302825	B244350	27-parameter
CD11b	BV510	ICRF44	BioLegend	301334	B244424	27-parameter
PD-1	BV480	EH12.1	BD	566112	8235507	27-parameter
CD103	BV421	Ber-ACT8	BioLegend	350214	B263432	27-parameter
CD8	BUV805	SK1	BD	564912	8127840	27-parameter
CD28	BUV737	Cd28.2	BD	564438	8236693	27-parameter
HLA-DR	BUV661	G46-6	BD	565073	7249926	27-parameter
CD4	BUV615	SK3	BD	624297	8191540	27-parameter
CD45RA	BUV563	HI100	BD	565702	8277539	27-parameter
CD3	BUV496	UCHT1	BD	564809	8107587	27-parameter
CD69	BUV395	FN50	BD	564364	8242749	27-parameter
CD45	PE-Cy7	HI30	BioLegend	304016	B264588	27-parameter
CD56	PE-CY5.5	CMSSB	eBioscience	35-0567-42	4348287	27-parameter
CD127	PE-CY5	eBioRDR5	eBioscience	15-1278-42	2025251	27-parameter
CX3CR1	PECF594	2A9-1	BioLegend	341624	B236542	27-parameter
GZMK	PE	GM6C3	Santa Cruz	sc-56125	E2214	27-parameter
CD3	FITC	HIT3a	BD	555339	7095651	Total RNAseq
CD8	BV786	RPA-T8	BD	563823	8080524	Total RNAseq
CD39	APC-Cy7	A1	BioLegend	328225	B231987	Total RNAseq
CD45RA	BV480	HI100	BD	566114	7160937	Total RNAseq
CD69	PE-Cy7	FN50	BD	557745	5205544	Total RNAseq
CD127	PE-Cy5	eBioRDR5	eBioscience	15-1278-42	2025251	Total RNAseq
CD103	BV421	Ber-ACT8	BioLegend	350214	B244354	Total RNAseq
CD25	BV711	BC96	BioLegend	302636	B242666	Tregs panel
CD127	PE-CY5	eBioRDR5	eBioscience	2021-12-23	2025251	Tregs panel
HLA-DR	BUV661	G46-6	BD	565073	7104603	Tregs panel
CD39	APC-Cy7	A1	Biolegend	328226	B60740	Tregs panel
ICOS	BUV395	DX29	BD	564777	7075884	Tregs panel
OX40	BV650	ACT35	BD	563658	8288968	Tregs panel
CD3	BUV496	UCHT1	BD	564809	8296578	Tregs panel
CCR8	BV421	433H	BD	566379	8214515	Tregs panel
IRF4	PE	3E4	eBioscience	BMS12-9858-80	4299177	Tregs panel
FOXP3	AF647	259D/57	BD	560045	6273737	Tregs panel
HLA-DR	APC-R700	G46-6	BD	565127	7076576	Cytokines panel
CD69	PE-Cy7	MOPC-21	BD	557745	5205544	Cytokines panel
CD3	FITC	UCHT1	BD	555339	7095651	Cytokines panel
IL-2	APC	MQ1-17H12	BD	554567	7110605	Cytokines panel
IFNγ	BV711	B27	BD	564039	7026849	Cytokines panel

TNFα	BUV395	MAB11	BD	563996	6224667	Cytokines panel
CD107a	BB630	H4A3	BD	624294	9345335	Cytokines panel

Supplementary Table 2. Characteristics of BC patients used for high-dimensional flow cytometry immunophenotyping

Characteristic	N (%)
All pts	54
Median age, years (range)	59,5 (32-90)
Surgery type	
Lumpectomy	31(57)
Mastectomy	23(43)
Hystology	
IDC	45 (83)
ILC	8 (15)
Other	1 (2)
pT	
1	13(24)
2	36 (67)
3	4 (7)
4	1 (2)
pN	
0	34 (63)
1	6 (11)
2	7 (13)
3	4 (7)
X	3 (6)
ER	
positive	47 (87)
negative	7 (13)
PgR	
positive	42 (78)
negative	12 (22)
HER2	27 (50)
0	
1+	5(9)
2+ FISH positive	3 (6)
2+ FISH negative	10(18)
3+	9(17)
Ki-67	
1-20%	22 (41)
>20%	32 (59)
LVI	
present extensive	13 (24)
present focal	9 (17)
absent	31 (57)
unknown	1 (2)

Supplementary table 3. Differentially expressed genes in CD127+ CD39lo vs CD127- CD39hi Trm CD8+ T cells

CD127+ CD39lo		
Ensembl gene IDs	Gene Symbol	FDR
ENSG00000168685.15	IL7R	0,001758864
ENSG00000161835.11	GRASP	0,045689295
ENSG00000139278.10	GLIPR1	0,021662031
ENSG00000126353.3	CCR7	0,045689295
ENSG00000074966.11	TXK	0,045689295
ENSG00000131459.13	GFPT2	0,033969175
ENSG00000126860.11	EVI2A	0,033969175
ENSG00000090382.6	LYZ	0,033969175
ENSG00000173391.9	OLR1	0,033969175
ENSG00000162676.12	GFI1	0,035841539
ENSG00000138378.19	STAT4	0,033969175
ENSG00000034053.14	APBA2	0,033969175
ENSG00000154127.10	UBASH3B	0,045689295
ENSG00000213445.10	SIPA1	0,043544845
ENSG00000107742.13	SPOCK2	0,033969175
ENSG00000075884.14	ARHGAP15	0,041419443
ENSG00000078589.13	P2RY10	0,033969175
ENSG00000115085.13	ZAP70	0,033969175
ENSG00000117091.10	CD48	0,033969175
ENSG00000169508.7	GPR183	0,033969175
ENSG00000101265.16	RASSF2	0,038829594
ENSG00000079335.20	CDC14A	0,043544845
ENSG00000105717.14	PBX4	0,035841539
ENSG00000170638.9	TRABD	0,045689295
ENSG00000134539.17	KLRD1	0,033969175
ENSG00000167895.14	TMC8	0,033969175
ENSG00000056558.11	TRAF1	0,033969175
ENSG00000173369.16	C1QB	0,045689295
ENSG00000198821.10	CD247	0,041534862
ENSG00000196154.12	S100A4	0,045689295
ENSG00000026025.16	VIM	0,033969175
ENSG00000121966.6	CXCR4	0,033969175
ENSG00000150637.9	CD226	0,045689295
ENSG00000118503.15	TNFAIP3	0,038829594
ENSG00000171310.11	CHST11	0,033969175
ENSG00000168918.14	INPP5D	0,033969175
ENSG00000010810.17	FYN	0,033969175
ENSG00000135074.15	ADAM19	0,045689295
ENSG00000091592.16	NLRP1	0,033969175
ENSG00000151883.18	PARP8	0,033969175
ENSG00000139112.11	GABARAPL1	0,045619308
ENSG00000124813.23	RUNX2	0,035669498
ENSG00000182568.17	SATB1	0,038829594
ENSG00000069667.16	RORA	0,033969175
ENSG00000158050.5	DUSP2	0,033969175
ENSG00000105122.13	RASAL3	0,035669498
ENSG00000162511.8	LAPTM5	0,033969175
ENSG00000184922.14	FMNL1	0,033969175
ENSG00000152495.11	CAMK4	0,045619308
ENSG00000110934.12	BIN2	0,033969175
ENSG00000090924.15	PLEKHG2	0,045689295
ENSG00000145819.18	ARHGAP26	0,033969175
ENSG00000157514.16	TSC22D3	0,038829594
ENSG00000172716.16	SLFN11	0,046851729
ENSG00000110848.8	CD69	0,042747554
ENSG00000154822.18	PLCL2	0,045689295
ENSG00000077150.20	NFKB2	0,035841539
ENSG00000136286.16	MYO1G	0,038737052
ENSG00000105329.10	TGFB1	0,033969175
ENSG00000059804.16	SLC2A3	0,033969175
ENSG00000145779.8	TNFAIP8	0,045689295
ENSG00000171522.6	PTGER4	0,033969175
ENSG00000141506.13	PIK3R5	0,045689295
ENSG00000196735.12	HLA-DQA1	0,045689295
ENSG00000115165.10	CYTIP	0,038829594

CD127- CD39hi		
Ensembl gene IDs	Gene Symbol	FDR
ENSG00000074410.14	CA12	0,045689295
ENSG00000116299.17	KIAA1324	0,045689295
ENSG00000113739.10	STC2	0,045689295
ENSG00000003989.17	SLC7A2	0,038829594
ENSG00000185630.18	PBX1	0,037942403
ENSG00000085733.16	CTTN	0,048797799
ENSG00000064651.14	SLC12A2	0,045689295
ENSG00000162989.5	KCNJ3	0,045689295
ENSG00000120549.18	KIAA1217	0,033969175
ENSG00000127954.12	STEAP4	0,043544845
ENSG00000150687.12	PRSS23	0,045689295
ENSG00000144218.19	AFF3	0,033969175
ENSG00000152661.9	GJA1	0,033969175
ENSG00000172403.11	SYNPO2	0,041534862
ENSG00000111057.11	KRT18	0,033969175
ENSG00000124766.7	SOX4	0,045689295
ENSG00000173848.19	NET1	0,033969175
ENSG00000134909.18	ARHGAP32	0,045689295
ENSG00000175356.13	SCUBE2	0,033969175
ENSG00000035403.17	VCL	0,035581052
ENSG00000167306.20	MYO5B	0,045689295
ENSG00000163697.17	APBB2	0,033969175
ENSG00000083307.12	GRHL2	0,035669498
ENSG00000160862.13	AZGP1	0,033969175
ENSG00000163435.16	ELF3	0,038829594
ENSG00000142192.21	APP	0,033969175
ENSG00000039068.19	CDH1	0,033969175
ENSG00000053747.16	LAMA3	0,045689295
ENSG00000061676.15	NCKAP1	0,038400194
ENSG00000261150.2	EPPK1	0,047069845
ENSG00000119888.10	EPCAM	0,038829594
ENSG00000041353.10	RAB27B	0,045689295
ENSG00000184292.7	TACSTD2	0,045689295
ENSG00000083857.14	FAT1	0,033969175
ENSG00000138771.16	SHROOM3	0,033969175
ENSG00000118513.19	MYB	0,045619308
ENSG00000104413.18	ESRP1	0,033969175
ENSG00000082175.15	PGR	0,033969175
ENSG00000130635.15	COL5A1	0,045689295
ENSG00000166145.14	SPINT1	0,035715039
ENSG00000054793.14	ATP9A	0,033969175
ENSG00000140443.15	IGF1R	0,033969175
ENSG00000117595.12	IRF6	0,045689295
ENSG00000196208.14	GREB1	0,033969175
ENSG00000128849.10	CGNL1	0,038829594
ENSG00000142949.17	PTPRF	0,033969175
ENSG00000185008.17	ROBO2	0,038829594
ENSG00000135862.6	LAMC1	0,033969175
ENSG00000172037.14	LAMB2	0,033969175
ENSG00000078114.18	NEBL	0,033969175
ENSG00000133026.12	MYH10	0,045619308
ENSG00000046604.13	DSG2	0,033969175
ENSG00000151892.14	GFRA1	0,038829594
ENSG00000166483.11	WEE1	0,038400194
ENSG00000124171.9	PARD6B	0,031307691
ENSG00000115112.8	TFCP2L1	0,045689295
ENSG00000170381.14	SEMA3E	0,043544845
ENSG00000125398.8	SOX9	0,033969175
ENSG00000164128.7	NPY1R	0,038829594
ENSG00000111799.21	COL12A1	0,033969175
ENSG00000108244.16	KRT23	0,045689295
ENSG00000117525.14	F3	0,044387716
ENSG00000134827.8	TCN1	0,045619308
ENSG00000182993.5	C12orf60	0,033969175
ENSG00000186340.15	THBS2	0,033969175

ENSG00000142347.19	MYO1F	0,038400194
ENSG00000076928.17	ARHGEF1	0,038829594
ENSG00000043462.12	LCP2	0,038829594
ENSG00000170571.12	EMB	0,035841539
ENSG00000113263.12	ITK	0,035669498
ENSG00000135905.19	DOCK10	0,034733814
ENSG00000133639.5	BTG1	0,045689295
ENSG00000101445.10	PPP1R16B	0,041419443
ENSG00000158805.12	ZNF276	0,045689295
ENSG00000184588.18	PDE4B	0,045689295
ENSG00000020633.18	RUNX3	0,035715039
ENSG00000125354.23	SEPTIN6	0,043544845
ENSG00000185477.5	GPRIN3	0,045689295
ENSG00000089820.15	ARHGAP4	0,045689295
ENSG00000104660.19	LEPROTL1	0,038829594
ENSG00000072818.12	ACAP1	0,045689295
ENSG00000168421.13	RHOH	0,047725875
ENSG00000164674.15	SYTL3	0,045689295
ENSG00000102760.13	RGCC	0,043544845
ENSG00000105639.18	JAK3	0,045689295
ENSG00000180353.11	HCLS1	0,038829594
ENSG00000048740.18	CELF2	0,045689295
ENSG00000137265.15	IRF4	0,045689295
ENSG00000081320.11	STK17B	0,045689295
ENSG00000186469.8	GNG2	0,045689295
ENSG00000100241.21	SBF1	0,045689295
ENSG00000114841.17	DNAH1	0,045689295
ENSG00000123329.19	ARHGAP9	0,045527888
ENSG00000198851.9	CD3E	0,045689295
ENSG00000170525.21	PFKFB3	0,041419443
ENSG00000136167.14	LCP1	0,045689295
ENSG00000127152.18	BCL11B	0,045619308
ENSG00000113448.19	PDE4D	0,045689295
ENSG00000058063.16	ATP11B	0,043544845
ENSG00000140368.13	PSTPIP1	0,048950799
ENSG00000102879.15	CORO1A	0,045689295
ENSG00000123146.20	ADGRE5	0,043165295
ENSG00000138670.17	RASGEF1B	0,045689295
ENSG00000164691.17	TAGAP	0,045689295
ENSG00000083457.12	ITGAE	0,045689295
ENSG00000100100.13	PIK3IP1	0,045689295
ENSG00000147251.15	DOCK11	0,045689295
ENSG00000185811.19	IKZF1	0,045689295
ENSG00000072786.13	STK10	0,048657751
ENSG00000082074.18	FYB1	0,045689295
ENSG00000122862.5	SRGN	0,045689295
ENSG00000118985.16	ELL2	0,045689295
ENSG00000153563.15	CD8A	0,045689295
ENSG00000116852.14	KIF21B	0,046607041
ENSG00000213949.10	ITGA1	0,045689295
ENSG00000143924.19	EML4	0,048657751
ENSG00000116824.5	CD2	0,049542759

ENSG00000104332.12	SFRP1	0,033969175

Supplementary Table 4. Characteristics of BC patients donating lymph node specimens

Characteristic	N (%)
All pts	13
Median age, years (range)	56 (38-82)
Surgery type	
Lumpectomy	6 (46)
Mastectomy	7 (54)
Hystology	
IDC	13 (100)
pT	
1	2 (15)
2	10 (77)
3	1 (8)
pN	
1	5 (38)
2	4 (31)
3	4 (31)
ER	
positive	10 (77)
negative	3 (23)
PgR	
positive	8 (62)
negative	5 (38)
HER2	
0	6 (46)
1+	1 (8)
2+ FISH positive	2 (15)
2+ FISH negative	2 (15)
3+	2 (15)
Ki-67	
1-15%	4 (31)
>15%	9 (69)
LVI	
present extensive	6 (46)
present focal	2 (15)
absent	5 (38)

Supplementary Table 5. Characteristics of BC patients used for RNA-seq experiment

Characteristic	N (%)
All pts	6
Median age, years (range)	50 (38-81)
Surgery type	
Lumpectomy	2 (33)
Mastectomy	4 (66)
Hystology	
IDC	4 (100)
pT	
1	2 (33)
2	4 (66)
pN	
0	4 (66)
1	2 (33)
ER	
positive	6 (100)
PgR	
positive	6 (100)
HER2	
0	2 (33)
1+	1 (17)
2+ FISH positive	1 (17)
2+ FISH negative	1 (17)
3+	1 (17)
Ki-67	
1-15%	2 (33)
>15%	4 (66)
LVI	
present extensive	3 (50)
absent	3 (50)
