# Supplementary material for: Single-cell profiling defines the prognostic benefit of CD39high tissue resident memory CD8+ T cells in luminal-like breast cancer
Source: Commun Biol. 2021 Sep 22;4:1117. doi: 10.1038/s42003-021-02595-z (PMC8458450; doi:10.1038/s42003-021-02595-z)
Supplement: Supplementary file 3 — Description of Additional Supplementary Files [file 42003_2021_2595_MOESM3_ESM.pdf]

Description of Additional Supplementary Files

File name: Supplementary Data 1.

Description: Source data underlying figures.

Supplementary Data 1a: Heatmaps Raw data available under accession code GSE114725.

Supplementary Data 1b,c: Frequency and MFI of each cell marker for each cluster, both for CD4 and CD8 T cells. Bar-plot data are reported as frequency of cells from tissues (tumor, normal or peritumor and blood) per cluster.

Supplementary Data 1d: Bar-plot data are reported as frequency of cells per cluster among Breast Cancer tumor samples divided as Luminal-A like , Luminal-B like and TN Breast Cancer or Hestrogen Receptor+ and Hestrogen Receptor-.

Supplementary Data 2b: MFI values of different cell markers among CD127+CD39lo, CD127-CD39lo and CD127- CD39hi CD8 subpopulations from tumor samples.

Supplementary Data 2c: Frequency of CD127+CD39lo, CD127-CD39lo and CD127- CD39hi CD8 subpopulations for tumor, peritumor and blood samples.

Supplementary Data 2d: Frequency of CD127- CD39hi Trm on CD8 cells among metastatic and non-metastatic lymphnodes.

Supplementary Data 2f: Expression of cell markers represented as frequency among CD127+CD39lo, CD127-CD39lo and CD127- CD39hi CD8 subpopulations.

Supplementary Data 3a: Heatmaps Raw data available under accession code GSE154842.

Supplementary Data 3b: Normalized Enrichment Scores Values for each gene signature obtained by gene set enrichment analysis (GSEA) of RNA-seq data.

Supplementary Data 3c,d: Raw data available under accession code GSE154842 .RNA-seq data and clinical data from METABRIC dataset were retrieved from cBioportal platform (http://www.cbioportal.org).

Supplementary Data 4a,b: Frequency and MFI of each cell marker for each cluster. Bar-plot data are reported as frequency of cells from tissues (tumor, normal or peritumor and blood) per cluster.

Supplementary Data 4d: MFI or frequency of cell markers among CD4+ Tconv, Teff and Tregs.

Supplementary Data 4f: Raw data available under accession code GSE154842 for Trm data and GSE128822 for Tregs data. RNA-seq data and clinical data from METABRIC dataset were retrieved from cBioportal platform (http://www.cbioportal.org).

Supplementary Data S1b: Frequency of CD4 and CD8 T cells per cluster from tumor, normal and blood tissues.

Supplementary Data S3b: RNA-seq data and clinical data from METABRIC dataset were retrieved from cBioportal platform (http://www.cbioportal.org).

Supplementary Data S3c,d: RAW data available under accession code GSE154842. RNA-seq data and clinical data from METABRIC dataset were retrieved from cBioportal platform (http://www.cbioportal.org).

Supplementary Data S4a: Frequency of CD4 and CD8 T cells per cluster from tumor used for Pearson correlation analysis.

Supplementary Data S4b: Frequency of Trm CD127-CD39 hi CD8 T cells and Tregs from tumor used for Spearman correlation.

Supplementary Data S4d: Frequency of CCR8+ ICOS+ and CCR8-ICOS- Tregs from tumor samples.

Supplementary Data S4e: Raw data available under accession code GSE154842 for Trm data and GSE128822 for Tregs data. RNA-seq data and clinical data from METABRIC dataset were retrieved from cBioportal platform (http://www.cbioportal.org)
