# Supplementary material for: Single-cell profiling defines the prognostic benefit of CD39high tissue resident memory CD8+ T cells in luminal-like breast cancer
Source: Commun Biol. 2021 Sep 22;4:1117. doi: 10.1038/s42003-021-02595-z (PMC8458450; doi:10.1038/s42003-021-02595-z)
Supplement: Supplementary file 5 — Reporting Summary [file 42003_2021_2595_MOESM5_ESM.pdf]

nature research

Corresponding author(s): Enrico Lugli, PhD

Last updated by author(s): Jul 7, 2020

Reporting Summary

Nature Research wishes to improve the reproducibility of the work that we publish. This form provides structure for consistency and transparency in reporting. For further information on Nature Research policies, see our Editorial Policies and the Editorial Policy Checklist.

Statistics

For all statistical analyses, confirm that the following items are present in the figure legend, table legend, main text, or Methods section.

n/a	Confirmed	
☐	☒	The exact sample size (*n*) for each experimental group/condition, given as a discrete number and unit of measurement
☐	☒	A statement on whether measurements were taken from distinct samples or whether the same sample was measured repeatedly
☐	☒	The statistical test(s) used AND whether they are one- or two-sided<br>*Only common tests should be described solely by name; describe more complex techniques in the Methods section.*
☐	☒	A description of all covariates tested
☐	☒	A description of any assumptions or corrections, such as tests of normality and adjustment for multiple comparisons
☐	☒	A full description of the statistical parameters including central tendency (e.g. means) or other basic estimates (e.g. regression coefficient) AND variation (e.g. standard deviation) or associated estimates of uncertainty (e.g. confidence intervals)
☐	☒	For null hypothesis testing, the test statistic (e.g. *F*, *t*, *r*) with confidence intervals, effect sizes, degrees of freedom and *P* value noted<br>*Give P values as exact values whenever suitable.*
☒	☐	For Bayesian analysis, information on the choice of priors and Markov chain Monte Carlo settings
☒	☐	For hierarchical and complex designs, identification of the appropriate level for tests and full reporting of outcomes
☐	☒	Estimates of effect sizes (e.g. Cohen's *d*, Pearson's *r*), indicating how they were calculated

Our web collection on statistics for biologists contains articles on many of the points above.

Software and code

Policy information about availability of computer code

Data collection

Flow cytometry data were collected using a FACSymphony A5 or an LSR Fortessa with FACSDiva Software Version 8.0.1 (BD Pharmingen).Sequencing data were collected using NextSeq 500/550 (Illumina). Unnormalized single-cell RNA-sequencing (scRNA-seq) counts from 8 breast cancers were downloaded from Gene Expression Omnibus dataset (GSE154842).

Data analysis

FACS: Flow Cytometry Standard (FCS) 3.0 files were analyzed with FlowJo 9 and 10 (FlowJo LLC). In dedicated experiments samples were further analyzed with Python version 3.7.3 using a custom-written script incorporating PhenoGraph retrieved from the scikit-learn package (https://github.com/luglilab/Cytophenograph). UMAP was obtained by UMAP Python package and visualized in FlowJo 10.

RNA-seq: Raw sequence data were quality-controlled using FastQC version 0.11.8 (http://www.bioinformatics.babraham.ac.uk/projects/fastqc). Single-end reads were aligned to the human genome (GENCODE Human Release 29; Reference genome sequence: GRCh38/hg38) using STAR version 2.5.1b. Alignments were performed using default parameters. Reads associated with annotated genes were counted with the STAR aligner option -quantMode geneCounts. Differential gene expression between human T cell subsets was assessed using the edgeR package (version 3.20.9) . Benjamini-Hochberg correction was applied to estimate the FDR.

Public data analyses: public data from Azizi et al., Cell, 2018 were used for the in silico sorting of T cell subset in Breast Cancer. Thresholds of expression were determined by calculating 25th percentiles of the mRNA distribution, by excluding cells of the lower percentile and cells double positive for CD4 and CD8A. Data were imported into R version 3.5.1 and analyzed with Seurat version 3.0.1 48. Genes detected in less than three cells or containing more than 200 features were excluded from the analysis. The resulting datasets were normalized and log-transformed using the “ScaleData” function in Seurat. Cluster analysis was performed by using the “FindClusters” function with a resolution of 1.2 and 1 for CD4+ and CD8+, respectively. DEGs were identified among clusters through the “FindAllMarkers” function in Seurat.
Gene Set Enrichment Analysis (GSEA) was performed using GSEA software (version 3.0; Broad Insitute, MIT) and gene list ranked based on log2 fold changes. The gene set enrichment analysis was conducted in pre-ranked mode with scoring scheme “classic” and 1,000 permutations. The maximum gene set size was fixed at 5,000 genes, and the minimum size fixed at 10 genes. The gene signature was

retrieved from the Molecular Signatures Database (MSigDB v6.2). (METABRIC) dataset was downloaded from the cBioPortal platform (http://www.cbioportal.org) 51. For the three signatures (CD127-CD39hi Trm; CD127+CD39lo Trm; CCR8hi ICOShi IRF4+ effector Treg), normalized signal values were converted into z-score. The median z-score value was used to classify tumor samples into Low and High expression groups. Survival analysis was performed with GraphPad Prism using the Kaplan-Meier (KM) approach and applying the Log-rank (Mantel-Cox) test to estimate survival curves comparison.

Other: Microsoft Excel version 15.37, GraphPad Prism version 7.0c and R software version 3.4.4

For manuscripts utilizing custom algorithms or software that are central to the research but not yet described in published literature, software must be made available to editors and reviewers. We strongly encourage code deposition in a community repository (e.g. GitHub). See the Nature Research guidelines for submitting code & software for further information.

Data

Policy information about availability of data

All manuscripts must include a data availability statement. This statement should provide the following information, where applicable:
- Accession codes, unique identifiers, or web links for publicly available datasets
- A list of figures that have associated raw data
- A description of any restrictions on data availability

Public available data were retrieved from Gene Expression Omnibus dataset (GSE114725). Gene sets of interes were retrieved from the Molecular Signatures Database (MSigDB v6.2).Transcriptomic and clinical data related to The Molecular Taxonomy of Breast Cancer International Consortium (METABRIC) dataset were downloaded from the cBioPortal platform (http://www.cbioportal.org). The bulk RNA-seq reported in this paper have been deposited in the Gene Expression Omnibus under accession code GSE147398.

Field-specific reporting

Please select the one below that is the best fit for your research. If you are not sure, read the appropriate sections before making your selection.

☒ Life sciences ☐ Behavioural & social sciences ☐ Ecological, evolutionary & environmental sciences

For a reference copy of the document with all sections, see nature.com/documents/nr-reporting-summary-flat.pdf

Life sciences study design

All studies must disclose on these points even when the disclosure is negative.

Sample size	Sample size was chosen taking into account the means of the target values between the different experimental groups, the standard error and the statistical analyses used. Additionally, the selection of sample size was based on previous studies conducted by this laboratory which allow for statistically valid comparisons.
Data exclusions	For total RNA-seq analysis, CD103+CD39lowCD127+, CD103+CD39hiCD127- and CD103-CD39-CD45RA-, pregated as Aqua- CD8+CD69+, were isolated from previously frozen tumor samples, with a FACSAria cell sorter. The sort procedure was performed on 6 different luminal-like BC samples and, based on the number of single cells obtained after sort of each population, 2 samples were excluded, because the minimum number of cells, suitable for subsequent total RNA-seq analysis, was not reached. For the analysis of effector molecule production we excluded one sample from the CD107 expression analysis due to faulty antibody staining.
Replication	All data were reliably reproduced in at least two independent experiments with the exception of total RNA-seq (once from 6 donors) and high-dimensional flow cytometry (once from 54 donors per tumor tissue, adjacent normal breast tissue with matched peripheral blood).
Randomization	For all the experiments, donors (BC patients) were randomly selected in order to avoid potential biases (see Supplementary Table 2-4 for more details). No manual randomization was performed on these samples.
Blinding	All the data were collected and analyzed in a non-blind fashion because did not involve subjective measurements.

Reporting for specific materials, systems and methods

We require information from authors about some types of materials, experimental systems and methods used in many studies. Here, indicate whether each material, system or method listed is relevant to your study. If you are not sure if a list item applies to your research, read the appropriate section before selecting a response.

Materials & experimental systems

n/a	Involved in the study
	[x] Antibodies
[x]	Eukaryotic cell lines
[x]	Palaeontology and archaeology
[x]	Animals and other organisms
	[x] Human research participants
	[x] Clinical data
	[x] Dual use research of concern

Methods

n/a	Involved in the study
[x]	ChIP-seq
	[x] Flow cytometry
[x]	MRI-based neuroimaging

Antibodies

Antibodies used

FLOW CYTOMETRY
27-paramter panel
Anti-human TCRgd - PerCP-Cy5.5 - B1 - BioLegend - Cat # 331224 - Lot # B260893
Anti-human NKG2A - FITC - REA110 - Miltenyi - Cat # 130-113-568 - Lot # 5190115118
Anti-human CD39 - APC-H7 - A1 - BioLegend - Cat # 328226 - Lot # B260740
Anti-human GZMB - APC-R700 - GB11 - BD - Cat # 560213 - Lot # 7339825
Anti-human TIGIT - APC - A15153G - BioLegend - Cat # 372706 - Lot # B259593
Anti-human CD25 - BV786 - 2A3 - BD - Cat # 741035 - Lot # 9011676
Anti-human CCR7 - BV711 - 150503 - BD - Cat # 566602 - Lot # 8248691
Anti-human OX40 - BV650 - ACT35 - BD - Cat # 563658 - Lot # 8288968
Anti-human CD161 - BV605 - HP-3G10 - Biolegend - Cat # 339916 - Lot # B245752
Anti-human CD27 - BV570 - O323 - BioLegend - Cat # 302825 - Lot # B244350
Anti-human CD11b - BV510 - ICRF44 - Biolegend - Cat # 301334 - Lot # B244424
Anti-human PD-1 - BV480 - EH12.1 - BD - Cat # 566112 - Lot # 8235507
Anti-human CD103 - BV421 - Ber-ACT8 - BioLegend - Cat # 350214 - Lot # B263432
Anti-human CD8 - BUV805 - SK1 - BD - Cat # 564912 - Lot # 8127840
Anti-human CD28 - BUV737 - Cd28.2 - BD - Cat # 564438 - Lot # 8236693
Anti-human HLA-DR - BUV661 - G46-6 - BD - Cat # 565073 - Lot # 7249926
Anti-human CD4 - BUV615 - SK3 - BD - Cat # 624297 - Lot # 8191540
Anti-human CD45RA - BUV563 - HI100 - BD - Cat # 565702 - Lot # 8277539
Anti-human CD3 - BUV496 - UCHT1 - BD - Cat # 564809 - Lot # 8107587
Anti-human CD69 - BUV395 - FN50 - BD - Cat # 564364 - Lot # 8242749
Anti-human CD45 - PE-Cy7 - HI30 - Biolegend - Cat # 304016 - Lot # B264588
Anti-human CD56 - PE-CY5.5 - CMSSB - eBioscience - Cat # 35-0567-42 - Lot # 4348287
Anti-human CD127 - PE-CY5 - eBioRDR5 - eBioscience - Cat # 15-1278-42 - Lot # 2025251
Anti-human CX3CR1 - PECF594 - 2A9-1 - Biolegend - Cat # 341624 - Lot # B236542
Anti-human GZMK - PE - GM6C3 - Santa Cruz - Cat # sc-56125 - Lot # E2214

Sorting for total RNA-seq
Anti-human CD3 - FITC - HIT3a - BD - Cat # 555339 - Lot # 7095651
Anti-human CD8 - BV786 - RPA-T8 - BD - Cat # 563823 - Lot # 8080524
Anti-human CD39 - APC-Cy7 - A1 - Biolegend - Cat # 328225 - Lot # B231987
Anti-human CD45RA - BV480 - HI100 - BD - Cat # 566114 - Lot # 7160937
Anti-human CD69 - PE-Cy7 - FN50 - BD - Cat # 557745 - Lot # 5205544
Anti-human CD127 - PE-Cy5 - eBioRDR5 - eBioscience - Cat # 15-1278-42 - Lot # 2025251
Anti-human CD103 - BV421 - Ber-ACT8 - BioLegend - Cat # 350214 - Lot # B244354

Tregs specific panel
Anti-human CD25 - BV711 - BC96 - BioLegend - Cat # 302636 - Lot # B242666
Anti-human CD127 - PE-CY5 - eBioRDR5 - eBioscience - Cat # 2021-12-23 - Lot # 2025251
Anti-human HLA-DR - BUV661 - G46-6 - BD - Cat # 565073 - Lot # 7104603
Anti-human ICOS - BUV395 - DX29 - BD - Cat # 564777 - Lot # 7075884
Anti-human OX40 - BV650 - ACT35 - BD - Cat # 563658 - Lot # 8288968
Anti-human CCR8 - BV421 - 433H - BD - Cat # 566379 - Lot # 8214515
Anti-human CD39 - APC-H7 - A1 - BioLegend - Cat # 328226 - Lot # B260740
Anti-human FOXP3-AF647-259D/57-BD-Cat#560045-Lot#6273737

Functionality panel
Anti-human HLA-DR-APCR700-G46-6-BD-Cat#565127-Lot#7076576
Anti-human CD69-PE-Cy7-MOPC-21-BD-Cat#557745-Lot#5205544
Anti-human CD3-FITC-UCTH1-BD-Cat#555339-Lot#7095651
Anti-human IL-2-APC-MQ1-17H12-BD-Cat#554567-Lot#7110605

	Anti-human IFNg-BV711-B27-BD-Cat#564039-Lot#7026849<br>Anti-human TNFa-BUV395-MAB11-BD-Cat#563996-Lot#6224667
Validation	All the reagents for flow cytometry used in this manuscript went through stringent validation steps as previously described (Mazza et al., Cytometry A., 2018; Brummelman et al., Nat. Protoc., 2019). Antibodies were all titrated to determine the optimal concentration. Flow cytometry antibodies were serially diluted as follows: 1:20, 1:40, 1:80, 1:160, 1:320, 1:640 and 1:1,280. Titration stainings included additional markers in order to validate the expression patterns on known cell subsets (e.g. T cells). Optimal concentration was defined by comparing the expression with other previously validated clones of a given antibody or with other previously validated lots of the same antibody clone. Any further information on the validation performed by the manufacturer can be retrieved from the manufacturers' websites.<br><br>Flow cytometry:<br>Fluorochrome-conjugated monoclonal antibodies were purchased from commercial vendors. High-dimensional flow cytometry was performed as previously described (Brummelman et al., Nat. Protoc., 2019).

Human research participants

Policy information about studies involving human research participants

Population characteristics	Samples from tumor tissue, breast gland normal tissue from the same surgical specimen and matched peripheral blood were used in this study. A total of 54 patients were included in the study. Age range: 32-90 years old.<br>Lymph nodes (either metastatic or tumor-free; n = 12 and 9, respectively) from Breast Cancer patients were included in the study. Total of patients: 13 median age 56 (38-82). None of the patients received neoadjuvant chemotherapy or endocrine therapy. Please, refer to Supplementary Table 2 and 3 for further details.
Recruitment	In this study were included patients affected by early stage BC, consecutively surgically treated (lumpectomy or mastectomy with sentinel lymph node biopsy and, if clinically indicated, axillary dissection) at Humanitas Cancer Center Breast Surgery Unit.
Ethics oversight	Humanitas Clinical and Research Center IRB approved sample and data collection) (Prot. No. Humanitas ONC-OSS-02-2017).

Note that full information on the approval of the study protocol must also be provided in the manuscript.

Clinical data

Policy information about clinical studies

All manuscripts should comply with the ICMJEguidelines for publication of clinical research and a completedCONSORT checklist must be included with all submissions.

Clinical trial registration	NA
Study protocol	Not a clinical trial
Data collection	Samples from Cancer patients were obtained in the operating room in a sterile field by the surgeon. Blood samples (12-15 mL; n = 54) were collected by venipuncture before anesthesia induction.
Outcomes	Outcomes were from the METABRIC consortium. Please refer to that repository for more information. No other outcomes were determined in the study. Histology of breast cancer specimens was determined by pathological examination of the tissue, according to international guidelines.

Dual use research of concern

Policy information about dual use research of concern

Hazards

Could the accidental, deliberate or reckless misuse of agents or technologies generated in the work, or the application of information presented in the manuscript, pose a threat to:

No	Yes	
☒	☐	Public health
☒	☐	National security
☒	☐	Crops and/or livestock
☒	☐	Ecosystems
☒	☐	Any other significant area

Experiments of concern

Does the work involve any of these experiments of concern:

No	Yes	
☒	☐	Demonstrate how to render a vaccine ineffective
☒	☐	Confer resistance to therapeutically useful antibiotics or antiviral agents
☒	☐	Enhance the virulence of a pathogen or render a nonpathogen virulent
☒	☐	Increase transmissibility of a pathogen
☒	☐	Alter the host range of a pathogen
☒	☐	Enable evasion of diagnostic/detection modalities
☒	☐	Enable the weaponization of a biological agent or toxin
☒	☐	Any other potentially harmful combination of experiments and agents

Flow Cytometry

Plots

Confirm that:

☒ The axis labels state the marker and fluorochrome used (e.g. CD4-FITC).

☒ The axis scales are clearly visible. Include numbers along axes only for bottom left plot of group (a 'group' is an analysis of identical markers).

☒ All plots are contour plots with outliers or pseudocolor plots.

☒ A numerical value for number of cells or percentage (with statistics) is provided.

Methodology

Sample preparation

In all assays, cells were stained for 15 min at room temperature with Zombie Aqua fixable viability dye (BioLegend) to exclude dead cells. Fluorochrome-conjugated monoclonal antibodies were purchased from BD Biosciences, BioLegend, eBioscience and Santa Cruz Biotechnology and titrated to determine optimal concentrations. Surface markers were evaluated by incubating cells at RT for 20 min. The Cytofix/Cytoperm kit (BD Biosciences) was used to detect intracellular cytokine expression on sorted T cell subsets. Measurement of transcription factors and intranuclear molecules was performed with FoxP3 Transcription Buffer Set (Invitrogen) or Transcription Factors Buffer Kit (BD) according to manufacturers' instructions.

Instrument

T cell subsets were FACS-sorted to purity by using a FACSAria III (BD Biosciences). All samples were acquired on FACSymphony A5 (equipped to detect 30 parameters).

Software

Flow cytometry and cell sorting:
Flow cytometry data were collected with FACSDIVA software and analyzed and compensated with FlowJo 9 and 10 (FlowJo LLC) by using single-stained controls prepared with antibody-capture beads (BD Biosciences).

High-dimensional flow cytometry data analysis:
Flow Cytometry Standard (FCS) 3.0 files were imported into FlowJo softwere version 9, and analyzed by standard gating to remove aggregates and dead cells, and identify CD4+ and CD8+ T cells. 1,000 of each CD8+ and CD4+ T cells per sample were subsequently imported in FlowJo version 10. Data were then biexponentially transformed and exported for further analysis in Python version 3.7.3 using a custom-made script (available at https://github.com/luglilab) that makes use of PhenoGraph from the scikit-learn package. Tissue samples were labeled with a unique computational barcode, converted into comma separated (CSV) files, and concatenated in a single matrix using the "concat" function in pandas (https://pandas.pydata.org/). The K value, indicating the number of nearest neighbors identified in the first iteration of the algorithm, was set to 1,000. UMAP applications were run in Python and visualized using FlowJo software version 10.
Clusters representing <1% were excluded from subsequent analyses. The data were then reorganized and saved as new FCS files, one for each cluster, that were further analyzed in FlowJo to determine the frequency of positive cells for each marker and the corresponding mean fluorescence intensity (MFI). These values were multiplied to derive the integrated MFI (iMFI, rescaled values from 10 to 100). The heatmap, showing the iMFI of each marker per cluster, and the subsequent metaclustering were performed using the gplots R package. Hierarchical metaclustering of all samples, based on the frequency of Phenograph clusters, was performed in R based on the Euclidean distance and Ward-linkage. Pearson correlation analysis was used to investigate the relationship between CD4+ and CD8+ clusters.
Hierarchical metaclustering of all samples, based on the frequency of Phenograph clusters, was performed in R based on the Euclidean distance and Ward-linkage.

Cell population abundance

Determined by standard flow cytometry gating

Gating strategy

Flow cytometric gating strategy for the isolation of Trm CD127-CD39hi and CD127+CD39lo populations is provided in Supplementary Figure 3. Briefly, lymphocytes were first selected on the basis of physical parameters and doublets and dead cells were excluded. Then CD8+ Trm were selected on the basis of CD69 and CD103 expression. Then we indified two subpopulations of Trm based on CD39 and CD127 expression. Flow cytometric gating strategy for Tregs identification is

provided in Supplementary Figure 4 and Figure 4. Lymphocytes were selected based on physical parameters and doublets and dead cells were excluded. Then CD4+ Tregs were selected on the basis of CD25 and CD127 and distinguished from CD4+ Tconv defined as CD25-CD127+ and CD4+ Teff defined as CD25-CD127-.

☒ Tick this box to confirm that a figure exemplifying the gating strategy is provided in the Supplementary Information.
